# Enhancer reprogramming in mammalian genomes

**DOI:** 10.1186/s12859-018-2343-7

**Published:** 2018-09-10

**Authors:** Mario A. Flores, Ivan Ovcharenko

**Affiliations:** Computational Biology Branch, National Center for Biotechnology Information, National Library of Medicine, National Institutes of Health, 8600 Rockville Pike, Bethesda, MD 20894 USA

**Keywords:** Enhancers, Evolution, Gene regulation, Transcription factor binding sites

## Abstract

**Background:**

Transcription factor binding site (TFBS) loss, gain, and reshuffling within the sequence of a regulatory element could alter the function of that regulatory element. Some of the changes will be detrimental to the fitness of the species and will result in gradual removal from a population, while other changes would be either beneficial or just a part of genetic drift and end up being fixed in a population. This “reprogramming” of regulatory elements results in modification of the gene regulatory landscape during evolution.

**Results:**

We identified reprogrammed enhancers (RPEs) by comparing the distribution of tissue-specific enhancers in the human and mouse genomes. We observed that around 30% of mammalian enhancers have been reprogrammed after the human-mouse speciation. In 79% of cases, the reprogramming of an enhancer resulted in a quantifiably different expression of a flanking gene. In the case of the Thy-1 cell surface antigen gene, for example, enhancer reprogramming is associated with cortex to thymus change in gene expression. To understand the mechanisms of enhancer reprogramming, we profiled the evolutionary changes in the TFBS enhancer content and found that enhancer reprogramming took place through the acquisition of new TFBSs in 72% of reprogramming events.

**Conclusions:**

Our results suggest that enhancer reprogramming takes place within well-established regulatory loci with RPEs contributing additively to fine-tuning of the gene regulatory program in mammals. We also found evidence for acquisition of novel gene function through enhancer reprogramming, which allows expansion of gene regulatory landscapes into new regulatory domains.

**Electronic supplementary material:**

The online version of this article (10.1186/s12859-018-2343-7) contains supplementary material, which is available to authorized users.

## Background

There has been a continuous interest in the study of regulatory evolution in mammals given that most phenotypic differences are hypothesized to result from regulatory differences [1]. In particular, distal cis-regulatory elements, such as enhancers, are fertile targets for evolutionary change [2]. Consequently, it is of fundamental importance to understand the mechanisms driving enhancer evolution. For example, it has been shown that morphological innovations are driven by the widespread emergence of new regulatory functions and these may arise through the modification of regulatory elements with ancestral roles [3–5]. Of particular interest are enhancers derived from a common ancestor that retain their function as enhancers but have changed their tissue-specificity during evolution. We have named this phenomenon enhancer reprogramming and refer to the regulatory elements in this category as reprogrammed enhancers (RPEs).

Several studies have addressed the forces governing the evolution of enhancers [2, 4, 6, 7], the repurposing of regulatory sequences [8], and the evolutionary innovation of transcription factor (TF) recognition sequences [6, 9]. However, the role of enhancer reprogramming in the evolution of the mammalian gene regulatory landscape is still largely unknown. Also unknown is the contribution of RPEs to gene regulatory changes. We need to emphasizes that our perspective to address this problem is different from the analysis of enhancer gains and losses between two mammalian species. We focused on the change in enhancer tissue-specificity during the mammalian evolution and identified a set of reprogrammed human and mouse enhancers. As the tissue-specificity of enhancers in the genome of the last common mammalian ancestor is unknown, we are not speculating whether the tissue-specificity of human or mouse enhancers is closer to the ancestral state. Additionally, many studies have addressed the problem of enhancer evolution from a gain/loss perspective. One example is a recent study that shows and validates experimentally the loss of the ZRS enhancer function which is a critical limb enhancer highly conserved across vertebrates [10]. Here we focus on those enhancers that show sequence conservation during evolution but that have been rewired in order to provide regulatory control in new distinct tissues.

In order to study RPEs, we took advantage of the growing number of high-throughput genome-wide maps of regulatory activity in the human and mouse genomes. Given that these organisms diverged relatively recently (approximately 65 to 75 million years ago [11]), a large fraction of orthologous enhancers could be identified reliably. It has been shown that 40% of the predicted mouse enhancers that have human orthologues are also predicted as enhancers in humans [8]. Thus, human and mouse genomes are excellent candidates for the study of enhancer reprogramming in mammals.

We identified genome-wide sets of RPEs from enhancer collections generated by the NIH Roadmap Epigenomics project [12] and the mouse ENCODE project [13]. We found that a high fraction of mammalian enhancers (42% in human and 24% in mouse) had been reprogrammed after the human-mouse speciation. In 79% of cases, the reprogramming of an enhancer resulted in quantifiably different expression of a flanking gene. For gene loci that include only one enhancer, the observed percentage of RPEs was significantly lower than expected by chance, which suggests that RPEs have an additive effect on transcriptional control of genes within well-established regulatory loci. By addressing the mechanisms that allow reprogramming of enhancers, we found that in 72% of cases, RPEs show an elevated density of newly acquired TFBSs suggesting that the main mechanism of enhancer reprogramming is the acquisition of new binding sites.

## Methods

### Enhancer predictions

We downloaded chromHMM segmentations (18 states) from the integrative analysis of 111 human epigenomes obtained by the NIH Roadmap Epigenomics project [12]. Next, we selected regions annotated as states 8 (EnhG2) and state 9 (EnhA1) as candidate human enhancers. We selected only these states because they are the only states with high levels of H3K4me1 and H3K27ac as well as low levels of H3K4me3 and, hence, the least likely to include false positives. For mouse, we downloaded candidate enhancers in 23 mouse tissues/cell types from the mouse ENCODE project [13] that were predicted based on a random forest classifier of histone marks [14], and, like human enhancers, exhibited high levels of H3K4me1 and H3K27ac, and low levels of H3K4me3. Since many enhancers predicted using histone marks may not have regulatory activity we verified that they show activity by overlapping them with experimentally verified enhancers from the VISTA Enhancer Browser [15]. We found that 71% (674/955) of human VISTA enhancers overlap human enhancers in at least one tissue. Similarly, 37% (214/615) of mouse VISTA enhancers overlap mouse enhancers defined by histone marks. The difference in the percentages is related to the number of tissues available for human (96) compared to mouse (23).

### Selection of matching tissues/cell types

We selected 11 pairs of orthologous tissues from the human and mouse datasets, which include 8 organs, one extremity, one tissue and one cell line referred to collectively as tissues, for simplicity (Table 1). The tissues were adult tissues with the exception of the embryonic mouse and human limb tissues. Also, we included a leukemia cell line that includes mouse erythroleukemia (MEL) and human immortalized myelogenous leukemia (K562).

**Table 1:** The number of human and mouse enhancers in 11 tissues. The table also includes the count of the three categories of enhancers in humans: enhancer gains (EGs), functionally conserved enhancers (FCEs), and reprogrammed enhancers (RPEs). BAT stands for brown adipose tissue. Leukemia refers to the human K562 cell line and mouse erythroleukemia. Limb refers to embryonic limb in human and limb e14.5 in mouse

Tissue	Human enhancers	Mouse enhancers	Human EGs	Human FCEs	Human RPEs
BAT	35,356	47,267	17,309	6566	11,481
Cortex	27,682	57,310	13,198	6070	8414
Heart	36,003	61,646	16,789	8370	10,844
Intestine	18,581	48,469	9296	3359	5926
Liver	37,241	53,162	21,061	6125	10,055
Lung	28,932	61,685	14,576	5890	8466
Placenta	31,221	61,926	17,925	5433	7863
Spleen	24,152	38,090	14,245	3102	6805
Thymus	14,722	35,854	7735	2362	4625
Limb	40,101	62,557	19,908	8501	11,692
Leukemia	19,907	36,488	12,797	1303	5807

### Data filtering

Since datasets of mouse enhancers consisted of peak locations that define the center of the region (mm9), we defined mouse enhancers as 1 kb regions centered on the center of a peak. Among human enhancers, we excluded those longer than 3 kbps, so-called stretch enhancers [16], which includes many super-enhancers [17]. Enhancer sets for 11 orthologous tissues in human and mouse were then filtered for repeats: regions with more than 75% repeats were removed.

All analyses based on intersecting genomic regions employed a minimum threshold of a 50 bps overlap.

### Categories of enhancers

Based on the sequence and function conservation of enhancers in the human and mouse genomes, enhancers were categorized as functionally conserved enhancers (FCEs), reprogrammed enhancers (RPEs) or enhancer gains (EGs). For this, we mapped enhancers between the human and mouse genomes and used the sets of axtNet human (hg19) to mouse (mm9) alignments pre-processed by the University of California, Santa Cruz (UCSC) with BLASTZ [18] and deposited at the UCSC Genome Bioinformatics Data web server [19].

To estimate the percentages of RPEs, FCEs and EGs in the human genome we used the following procedure. First, human enhancers were mapped to the mouse genome (and vice a versa). Enhancers that did not align were categorized as EGs. Second, enhancers and their orthologous regions were overlapped with the tissue-specific enhancers of the 11 tissues in human and mouse, respectively. Cases where both the enhancer and the orthologous region overlapped same tissues were considered FCEs. However, if there was at least one case where the orthologous region overlapped mouse enhancers in a tissue, in which the human enhancer is not active, then the enhancer was considered a RPE. Finally, the remaining enhancers were considered EGs.

To categorize enhancers for a pair of tissues, we followed the next procedure. For each pair of tissues (A and B) in human, the subsets of non-overlapping enhancers were selected (${A}_1^H$ and ${B}_1^H$). Sets of non-overlapping enhancers were also selected for mouse orthologous tissues to produce subsets (${A}_1^M$ and ${B}_1^M$). Next ${A}_1^H$ and ${B}_1^H$ were aligned to the mouse genome to produce subsets (${A}_2^H$ and ${B}_2^H$). Enhancers that did not align were labeled as EGs in each human tissue. Next, we overlapped enhancers (${A}_2^H\cap {A}_1^M$) and labeled them FCEs in tissue A and for ${B}_2^H\cap {B}_1^M$ as FCEs in tissue B. Mouse enhancers that did not overlap in the previous step were separated as disjoint subsets ${A}_2^M$ and ${B}_2^M$. Next, we overlapped ${A}_2^H\cap {B}_2^M$ which resulted in the set of enhancers reprogrammed to mouse tissue B and human tissue A while ${B}_2^H\cap {A}_2^M$ in the set of enhancers reprogrammed to mouse tissue A and human tissue B. Enhancers not overlapped in the previous step were joined with the subset of EGs.

The hierarchically-clustered heatmap (Additional file 1: Figure S2) was generated using the Seaborn visualization library based on matplotlib [20]. Clusters were calculated using the UPGMA algorithm [21].

### Gene expression enrichment

RNA-Seq data were downloaded from the Roadmap Epigenomics project [12] and the mouse Encode project [13] for the available 7 of the 11 matching tissues / cell types: heart, liver, cortex, spleen, thymus, lung and intestine. Gene expression was normalized by the median value of expression for all genes in a tissue. A gene locus boundary was defined as half the distance between the end of a gene and the start of the consecutive gene.

To quantify if the reprogramming of enhancers is reflected in changes of the level of gene expression we used the following procedure. For each pair of tissues in a reprogramming case (mouse tissue A and human tissue B), the genes that include RPEs within their loci were selected and their expression values in tissue B obtained and compared to a control. The control consisted of the expression values of the genes from the human tissue A. We addressed if the expression of the genes in the tissue B was higher than the expression in the tissue A. For this we calculated a Wilcoxon rank sum test *p*-value.

### Comparison of overrepresented TFBSs between RPEs and FCEs

To determine if enhancer reprogramming to the mouse tissue A and the human tissue B is driven by changes in the composition of TFBS, we implemented the following procedure. First, a library of TFBS was downloaded from the MEME database [22]. This library combines Eukaryote DNA [23], JASPAR [24], CIS-BP [25], and HOCOMOCO [26] libraries of TFBSs and includes 4004 individual TFBSs. We extracted a non-redundant subset of 1431 TFBS and used it to scan for occurrences of motifs in human and mouse genomes using the FIMO tool [27]. The tissue-specific TFBS enhancer composition was established by identifying TFBSs overrepresented in each set of FCEs (tissue-TFBSs). For this, we scanned for TFBSs within FCEs regions and calculated *p*-values using a Poisson distribution with Bonferroni correction for multiple testing against control regions. The controls consisted of random regions matched for length, GC and repeat content.

To determine if enhancer reprogramming to the mouse tissue A and the human tissue B is driven by a change in TFBS enhancer composition specific to the tissue A to tissue B transfer, we found overrepresented TFBSs in RPEs in the tissue B using the procedure described in the previous paragraph first. Next, the number of overrepresented TFBSs of RPEs that were also present in the tissueB-TFBSs was calculated and the percentage of overlap obtained. A control was generated by calculating the percentage of overrepresented TFBS of RPEs that were also present in the set of tissueA-TFBSs.

Using human-mouse genome alignments, described above, we compared the distribution of TFBSs in human and mouse orthologs of RPEs and FCEs. Differences and similarities in TFBS distributions were classified as conserved sites (TFBSCs), reshuffled sites (TFBSHs), gained sites (TFBSGs), and reused sites (TFBSRs). TFBSCs are the sites that can be mapped between the human and mouse enhancers bound by same TFs, TFBSHs are the sites that can’t be mapped, however they are present in a human and mouse enhancer and they are bound by the same TF, TFBSGs are the sites present in a human enhancer but not in the mouse orthologue counterpart and TFBSRs are the sites that can be mapped between human and mouse, however mutations within these sites had changed the TFBS motif resulting in the binding of distinct TFs. For each of these categories, the TFBS density was computed and compared between RPEs and FCEs (Fig. 4b). For every pair of enhancers (reprogrammed to the mouse tissue A and the human tissue B), the density of TFBSCs, TFBSHs, TFBSGs and TFBSRs was calculated. For this, we scanned the tissue-specific TFBS of the tissue B human RPEs and the tissue-specific TFBS of the tissue A mouse RPEs counterparts. Next, we aligned the pairs of regions and calculated the density of the four categories of sites in the RPEs of the tissue B. Controls were generated by calculating the density of the four categories of sites in FCEs of the tissue B. Next, the TFBS density in RPEs was categorized as either (i) higher than in FCEs, (ii) lower than in FCEs or (iii) equal to the FCE TFBS density.

## Results

### Extensive enhancer reprogramming in mammals

**Fig. 1:**
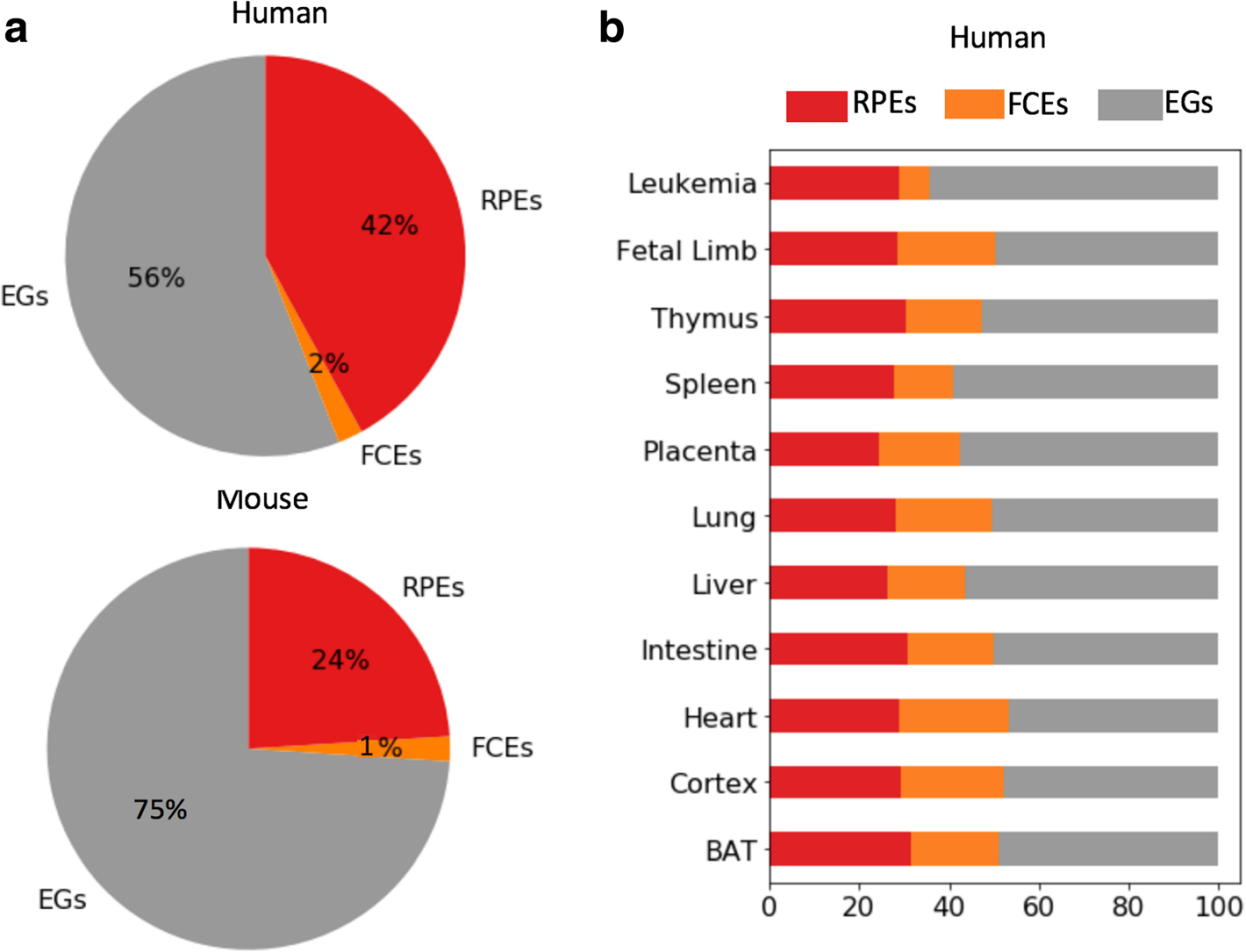
Reprogrammed enhancers are prevalent in mammalian genomes. **a.** Average percentage of reprogrammed enhancers (RPEs), functionally conserved enhancers (FCEs) and enhancer gains (EGs) in the human genome. **b.** Proportion of the 3 categories of enhancers per human tissue

There are 164,253 and 236,829 enhancers in the human and mouse genomes, respectively, that can be assigned to one of the 11 matching tissues in these two species (Table 1; see Methods for details). The sets of predicted enhancers in this study were obtained from the chromHMM segmentations of the human and mouse genomes computed using a large set of histone marks [12, 13]. An analysis of sequence and function conservation of these human and mouse enhancers showed that 2% of the human enhancers are conserved with mouse at the sequence level and are active in the same set of tissues (FCEs or functionally conserved enhancers). Fifty-six percent of human enhancers are not conserved with mouse and represent enhancer gains (EGs) while the remaining 42% are conserved with mouse at the sequence level but are active in a partially/fully different set of tissues. We named the latter set reprogrammed enhancers (RPEs) (Fig. 1a). The breakdown of mouse enhancers into the FCE, EG, and RPE categories is 1%, 75%, and 24%, respectively, with the difference in human and mouse category breakdowns reflecting the difference in the number of enhancers identified in these genomes.

The cumulative enhancer reprogramming rate obtained comparing all mouse tissues with a specific human tissue, defined as the percentage of enhancers that were categorized as reprogrammed, is relatively uniform across tissues (Table 1, Fig. 1) with the minimum of 25% (7863 RPEs out of 31,221 enhancers) enhancers reprogrammed to human placenta and the maximum of 30% (8414 RPEs out of 27,682 enhancers) of enhancers reprogrammed to human cortex (Table 1, Fig. 1b). We speculate that placenta may show the lowest proportion of RPEs (25%) and a high proportion of EGs (57%) in agreement with the finding that the mammalian placenta is remarkably different between species [28]. For individual pairs of tissues, the enhancer reprogramming rate has a minimum of 4.4% enhancers reprogrammed to mouse thymus and human placenta and a maximum of 11% of enhancers reprogrammed to mouse heart and human limb (Additional file 1:Figure S2).

Our estimate of the percentage of reprogrammed enhancers while substantial might be rather conservative, as availability of enhancer data from additional tissues and/or species will reveal additional RPEs in the current set of EGs or FCEs.

### Enhancer reprogramming leads to altered gene expression

To address if the change in the function of RPEs is reflected in the expression of their target genes, we selected seven tissues for which RNA-Seq data were available for both mouse and human (see Methods). Starting with the set of RPEs active in mouse liver and human heart, we obtained expression values for their flanking genes. We found that the median expression of genes flanking these RPEs is 1.4-fold higher in human heart than in human liver (*p*-value = 2.1e-5, Wilcoxon rank sum test). Similarly, the expression of mouse genes flanking these RPEs is 1.7-fold higher in mouse liver than in mouse heart (*p*-value = 2.8e-4, Wilcoxon rank sum test). We note that comparisons were made between two human tissues (heart and liver) and, separately, between two mouse tissues (liver and heart). We repeated this procedure for 42 sets of RPEs and observed a change in gene expression matching the change in enhancer activity for 33 of them (79%) (*p*-value = 0.04, Fisher’s exact test). As control, we repeated the above analysis for human heart FCEs and, as expected, observed greater expression of their proximal genes in human heart than in human liver (a 2.8-fold enrichment). Similarly, for mouse liver FCEs there was a greater expression of proximal genes in mouse liver compared to mouse heart (a 3.3-fold enrichment). On the basis of this finding, our results suggest that reprogramming of enhancers often leads to a concordant and significant reprogramming of their target genes.

To identify examples of likely enhancer reprogramming, we focused on gene loci that contained a single human RPE in a tissue pair in order to reduce the possibility of other enhancers controlling the gene. An interesting candidate RPE is the enhancer that is 9 kbs upstream of the Thy-1 cell surface antigen (THY1) gene. THY1 is a member of the immunoglobulin gene superfamily. This and other GPI-linked molecules have been implicated in key developmental events including selective axonal fasciculation and highly specific growth and innervation of target tissues [29]. Consistent with reprogramming, we found that the expression of THY1 is significantly higher in human cortex than human thymus (a 21.5-fold difference), while in mouse, in contrast, the trend is reversed (3.7-fold higher in thymus) (Additional file 1: Figure S3). This is corroborated by previous reports, where it has been shown that THY1 is expressed in mouse thymocytes and peripheral T cells and, thus, has been widely used as a T cell marker in mouse thymus [30]. In humans, however, THY1 is only expressed in neurons [31]. The basis of this altered tissue specificity has been hypothesized to be the differential presence of an Ets-1 binding site in the third intron of the gene [30]. However, as mentioned in that report, their experiments did not test specifically for regulatory sequences in the 5′ flanking sequences [32] where we found the RPE (Additional file 1: Figure S5).

### RPEs contribute to the regulation of genes within multi-enhancer loci

**Fig. 2:**
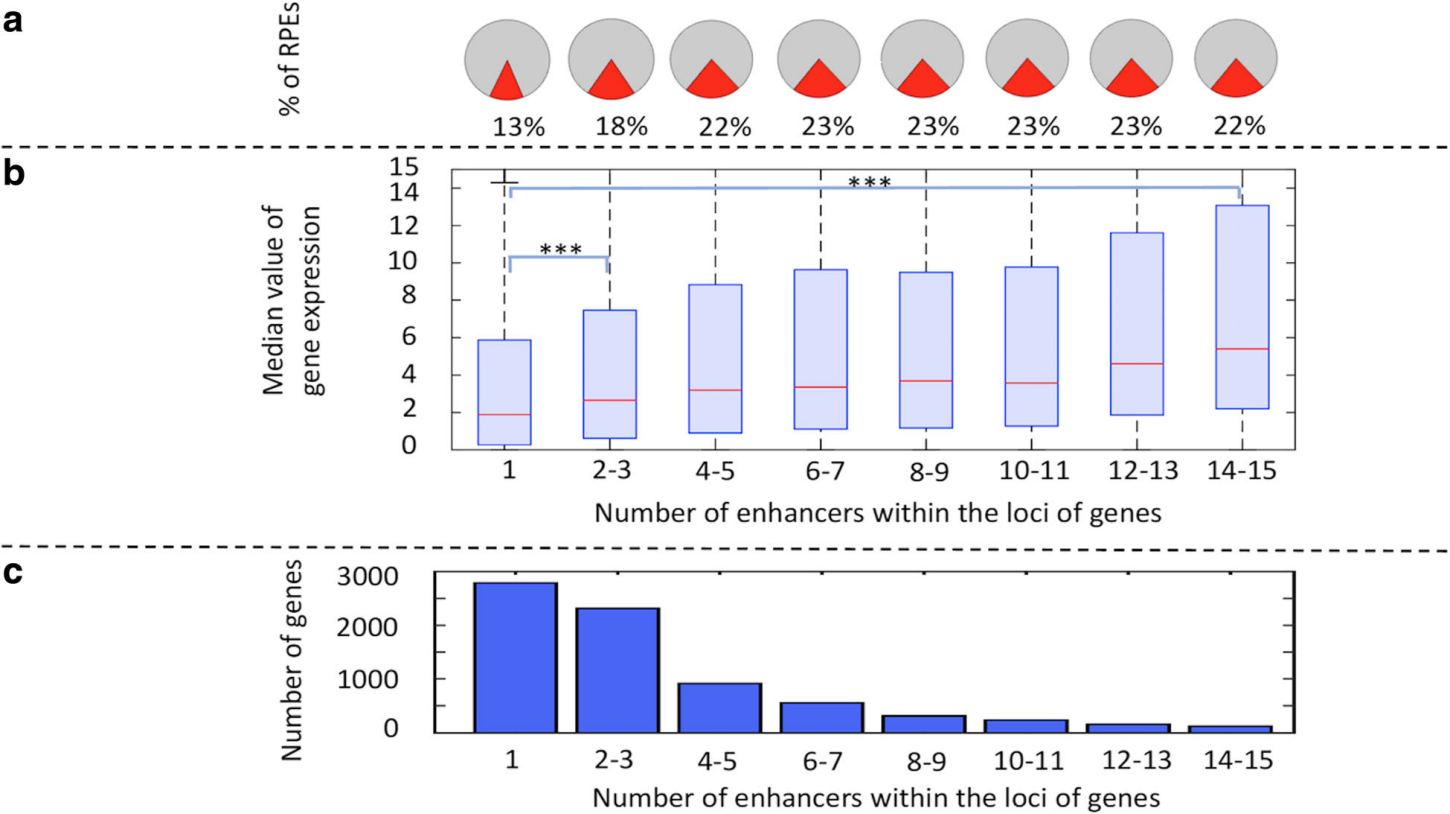
RPEs in multi-enhancer loci (reprogrammed to mouse liver and human heart). Gene loci were binned by the number of enhancers in a locus (x-axis). **a**. Proportion of RPEs in the set of locus enhancers. **b.** Median value of gene expression. (*** refers to a *p*-value < 0.0001.) **c.** The histogram of gene counts

**Fig. 3:**
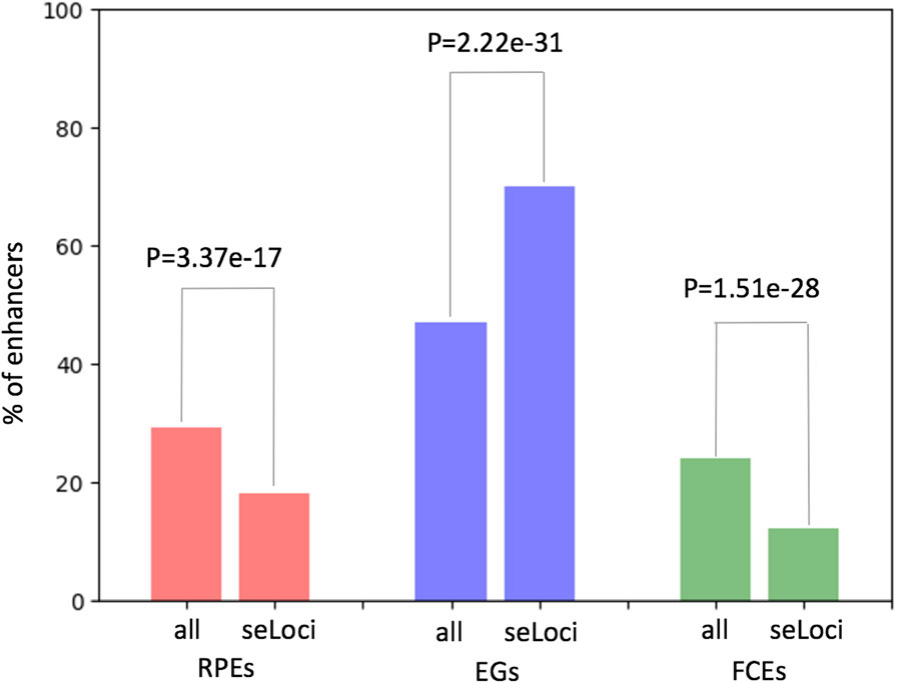
Enhancer distribution in seLoci and regular gene loci. The percentage of RPE, EG, and FCE enhancers in gene loci that contain only one enhancer (seLoci) or any number of enhancers (all). The *p*-values were calculated using the Fisher’s exact test

We next examined the contribution of RPEs to gene regulation in multi-enhancer loci (Fig. 2, Additional file 1: Figure S4a). For this, we calculated the median value of gene expression with genes binned by the number of enhancers within the loci of genes in human heart (Fig. 2b) and, in each bin, calculated the percentage of enhancers categorized as RPEs (Fig. 2a). We selected human heart as an example because several studies had reported the need for additional studies to delineate the differences in molecular mechanisms of mouse models of human heart and our study of enhancer reprogramming could contribute by providing data on those regulatory regions that may have changed their function during evolution [31, 33]. We found a positive correlation between the number of enhancers in a gene locus and the proportion of those categorized as RPEs. Also, we observed a known positive correlation between the expression level of genes and the number of enhancers in a gene locus [34]. However, there seems to be a limit in the increase of the expression level of genes related to the number of enhancers within their loci. We found that for loci with more than 15–20 enhancers, the expression level stabilizes. We also found that for gene loci that include only one enhancer (seLoci) (Additional file 1: Figure S4b), the observed percentage of RPEs is significantly lower than expected by chance (Methods, Fig. 3). We found a similar trend for FCEs, while the trend was opposite for EGs (Fig. 3).

We repeated the analysis for two tissues that had also been used in numerous mouse models (liver and lung) (Additional file 1: Table S2 and Table S3) and found similar results. This indicates that RPEs are disproportionately located within the loci of genes that contain multiple enhancers. The percentage of RPEs in a pool of locus enhancers increases with the number of enhancers within the locus (Fig. 2a and c).

These results suggest that enhancer reprogramming primarily plays a role in regulating gene expression by fine-tuning gene expression in established gene loci (those that already contain multiple active enhancers).

### Changes in the TFBS composition underlie enhancer reprogramming

To determine if enhancer reprogramming is driven by changes in the composition of TFBS, we implemented a procedure (see Methods) where we first established the tissue-specific TFBSs composition in a human tissue by identifying TFBSs overrepresented in FCEs in that tissue. Next, we generated a list of overrepresented TFBS in human RPEs (see Methods). To quantify the changes of TFBS composition, we calculated the percentage of overlap of the list of RPE TFBSs with the list of FCE TFBSs. For control, we overlapped the list of RPE TFBSs with the list of tissue-specific TFBSs in a second tissue. If the reprogramming of enhancers has been driven by changes in the composition of TFBSs within RPEs, then we should observe a significant overlap with FCE TFBSs compared to the control.

**Fig. 4:**
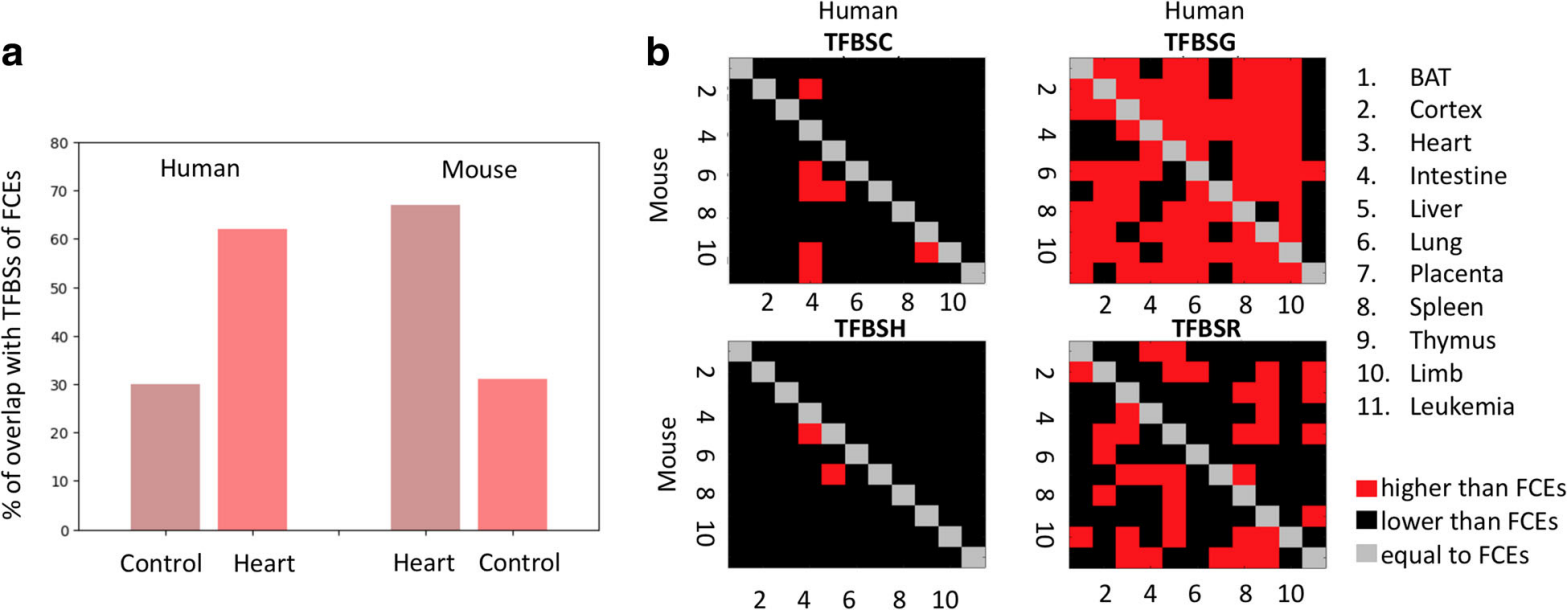
TFBS composition of RPEs and FCEs. **a.** Percentage of TFBSs overrepresented in RPEs, which are also overrepresented in FCEs. Cases for enhancers reprogrammed to mouse tissues and human heart. Controls (liver) are shown for comparison. **b.** Comparison of TFBS densities for four categories of sites, conserved (TFBSC), gained (TFBSG), reshuffled (TFBSH), and reused (TFBSR), for 110 cases of enhancer reprogramming. The densities of sites were calculated for the four categories of sites of RPEs normalized to densities of sites in FCEs. The diagonal indicates the densities of FCEs since RPEs are not defined for the same tissue in two species. For each plot, the top-right corner corresponds to evolutionary changes between the mouse and human genomes with the human genome as a reference. In the case of the bottom-left corners, the reference is the mouse genome

Using 11 cases of reprogramming to one of the mouse tissues and human heart, we found that the overlap of RPE TFBSs with FCE TFBSs of human heart is between 60 and 72% with the exception of the mouse leukemia cell, in which it was only 42%. In contrast, the overlap with controls was only between 21 and 32% (Fig. 4a). In the complementary case with reprogramming to mouse heart, we observed similar results, namely a 67–71% range for enhancers reprogrammed to mouse heart versus 32–35% for controls. These results suggest that the change in the function of RPEs is driven primarily by changes in the composition of TFBSs. For example, in the case of enhancers (reprogrammed to mouse liver and human heart), we observed a 1.1-fold depletion in TFBSs of hepatocyte nuclear factor 4 (HNF4A), a key TF involved in liver development [35], accompanied by a 1.5-fold enrichment of TFBSs of myocyte enhancer factor 2A (MEF2A), a key TF involved in heart development [36], when comparing human and mouse counterparts of these RPEs.

Next, we investigated the mechanisms underlying the changes of TFBSs within RPEs. For this, we established four categories of TFBSs, namely, conserved sites (TFBSCs), reshuffled sites (TFBSHs), gained sites (TFBSGs), and reused sites (TFBSRs), based on their alignment between the human and mouse counterpart enhancer regions (see Methods). We found that RPEs feature a greater density of TFBSGs as compared to FCEs in 73% of tissue pairs (80/110) (Fig. 4b). The density of TFBSCs and TFBSHs is lower in RPEs than in FCEs in 94% and 98% of cases, respectively. The density of TFBSRs doesn’t display a specific trend in comparison of FCEs with RPEs. These results argue for the evolutionary conservation of TFBSs in FCEs, which might have been expected given the functional conservation of the function of these sequences in contrast to the rapid change of the TFBS composition in enhancers being reprogrammed. RPEs mainly change their TFBS landscape through acquisition of new TFBSs accompanied by loss of original active TFBSs and not through reuse of active TFBSs. This suggests that the positions of active TFBSs within an enhancer are not nearly as important as the overall TFBS composition of an enhancer, i.e., the whole sequence of enhancers being reprogrammed is used for innovation consisting of TFBS loss and gain occurring at different enhancer positions.

For example, in the case of the previously described THY1 gene hosting a single RPE (Additional file 1: Figure S6a), there are two TFBSRs and four TFBSGs (Additional file 1: Figure S6b). Gained sites include TFBSs for transcription factors Ewing Sarcoma protein (EWS) and protein atonal homolog 1 (ATOH1). EWS is part of the FET family of DNA and RNA binding proteins, which has been implicated in brain development [37]. ATOH1 is a transcription factor of the NOTCH pathway, a key regulator of cerebellar development. Thus, 4 of 6 (67%) tissue-specific TFBS within the enhancer of THY1 are new and associated with brain expression, consistent with the idea that the main mechanism of reprogramming is acquisition of new sites for TFs that are specific to a new tissue [38]. The reused sites in the THY1 reprogrammed enhancer are both EWS BS rewired from sites for MYF5 in the mouse sequence. MYF5 is associated with the development of thymic myeloid cells [39]. This suggests that a secondary mechanism of reprogramming may be a reuse of a TFBS after mutations have rewired the site for a TF suited to the new tissue. Together, these results agree with a model dominated by TFBSGs and assisted by TFBSRs within a regulatory element altering the function of that regulatory element and its tissue-specificity.

## Conclusions

There are still many open questions in the study of the evolution of the mammalian gene regulatory landscape. Here, we provide some insight into the role of enhancer reprogramming in the evolution of the mammalian gene regulation.

First, we find that approximately 30% of mammalian enhancers have been reprogrammed since the mouse-human speciation, demonstrating that enhancer reprogramming is a prevalent phenomenon. A similar result was obtained in a comprehensive comparative analysis of the mouse and human DNase I hypersensitive sites (DHS) across multiple tissues [6]. The authors of that study showed that approximately 36% of DHSs evolutionary conserved between human and mouse have undergone repurposing (which we refer to as reprogramming). As DHSs represent areas of accessible chromatin and not necessarily regulatory elements, our study provides a focus on enhancers and the reprogramming of the gene regulatory landscape complimentary to the original study.

Second, we show that in 79% of cases, the reprogramming of an enhancer resulted in a quantifiably different expression of a flanking gene, which provides evidence of the change of function of RPEs.

Third, we found that only 4% of RPEs are located within the loci of genes that contain a single enhancer, suggesting that RPEs are mainly located within well-established regulatory loci. In contrast, there is a significantly higher proportion (11%) of EGs located within loci that include only one enhancer.

Fourth, we confirm that there is a positive correlation between the expression level of a gene and the number of its enhancers (11). However, we also find that there is a limit in the number of enhancers that can additively increase expression levels. Once this limit is reached (at approximately 17–20 enhancers), expression stabilizes.

Fifth, we find that the percentage of RPEs within multi-enhancer loci increases with a higher number of enhancers. Given the link between the number of enhancers within the locus of a gene and its expression levels, this suggests that RPEs may additively fine-tune the expression of genes.

Finally, we show that RPEs are mainly established through gains and losses of TFBSs, not reuse/reprogramming of active TFBSs. While the previously referred study of DHS reprogramming showed that enhancer repurposing is associated with tissue-specific TF binding sites changes, we categorized these changes as conserved, reshuffled, gained and reused. We show that the main mechanism of enhancer reprogramming took place primarily through the gain and loss of TFBSs (72% of cases) and not reuse of active TFBSs, as might be assumed. Similar results for a single TF were found in an experimental study of the evolutionary rewiring of the transcriptional master regulator p63 in mouse and human keratinocytes. The authors of that study found that 75% of the p63 target sites could mostly be attributed to evolutionary gains/losses while 25% are conserved [40]. In agreement with the Sethi’s study, we found that between 66 and 82% of predicted sites are categorized as gained sites while 16–22% are conserved sites depending on the TF. However, our approach allows profiling multiple TFs enriched in tissue-specific enhancers and identify differences between different classes of TFs. In addition, our results quantify the differences in gene expression for loci with increasing number of RPEs which correlates with increasing number of TFBSs (Fig. 2). In summary, our results are in agreement with Sethi et al. and also generalize the effects of multiple gained, lost, and conserved TFBSs within RPEs and thus extending the study to an analysis of the evolutionary rewiring of regulatory elements.

In summary, our study reports a widespread enhancer reprogramming in mammals and suggests that enhancer reprogramming has been a key component of adaptation of mammalian regulatory landscapes.

## Additional file

Additional file 1: Supplementary materials. (PDF 2887 kb)

## Abbreviations

BAT: Brown adipose tissue
EG: Enhancer gain
FCE: Functionally conserved enhancer
MEL: Mouse erythroleukemia
RPE: Reprogrammed enhancers
TFBS: Transcription factor binding site
TFBSC: Conserved transcription factor binging site
TFBSG: Gained transcription factor binging site
TFBSH: Reshuffled transcription factor binging site
TFBSR: Reused transcription factor binging site
