# Supplementary material for: Enhancer reprogramming in mammalian genomes
Source: BMC Bioinformatics. 2018 Sep 10;19:316. doi: 10.1186/s12859-018-2343-7 (PMC6131754; doi:10.1186/s12859-018-2343-7)
Supplement: Supplementary file 1 — Supplementary materials. (PDF 2887 kb) [file 12859_2018_2343_MOESM1_ESM.pdf]

Supplementary Materials

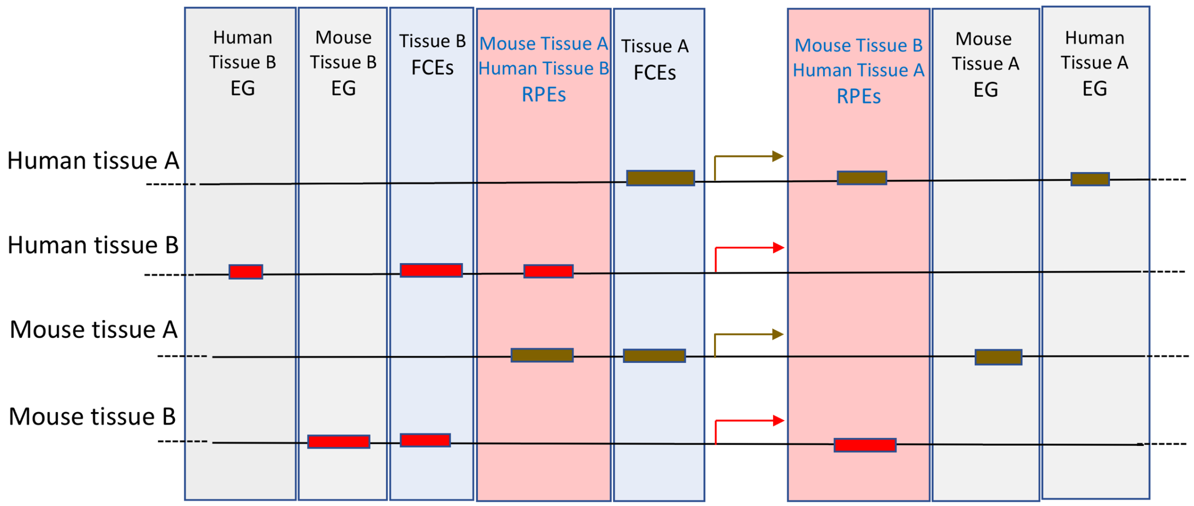

Figure S1. Schematic of reprogrammed enhancers. Human enhancers and their mouse counterparts were categorized based on their sequence alignment as: functionally conserved enhancers (FCEs) present in both species, reprogrammed enhancers (RPEs) present in both species but differing in tissue/cell-specificity, and enhancer gains (EGs) present in one species.

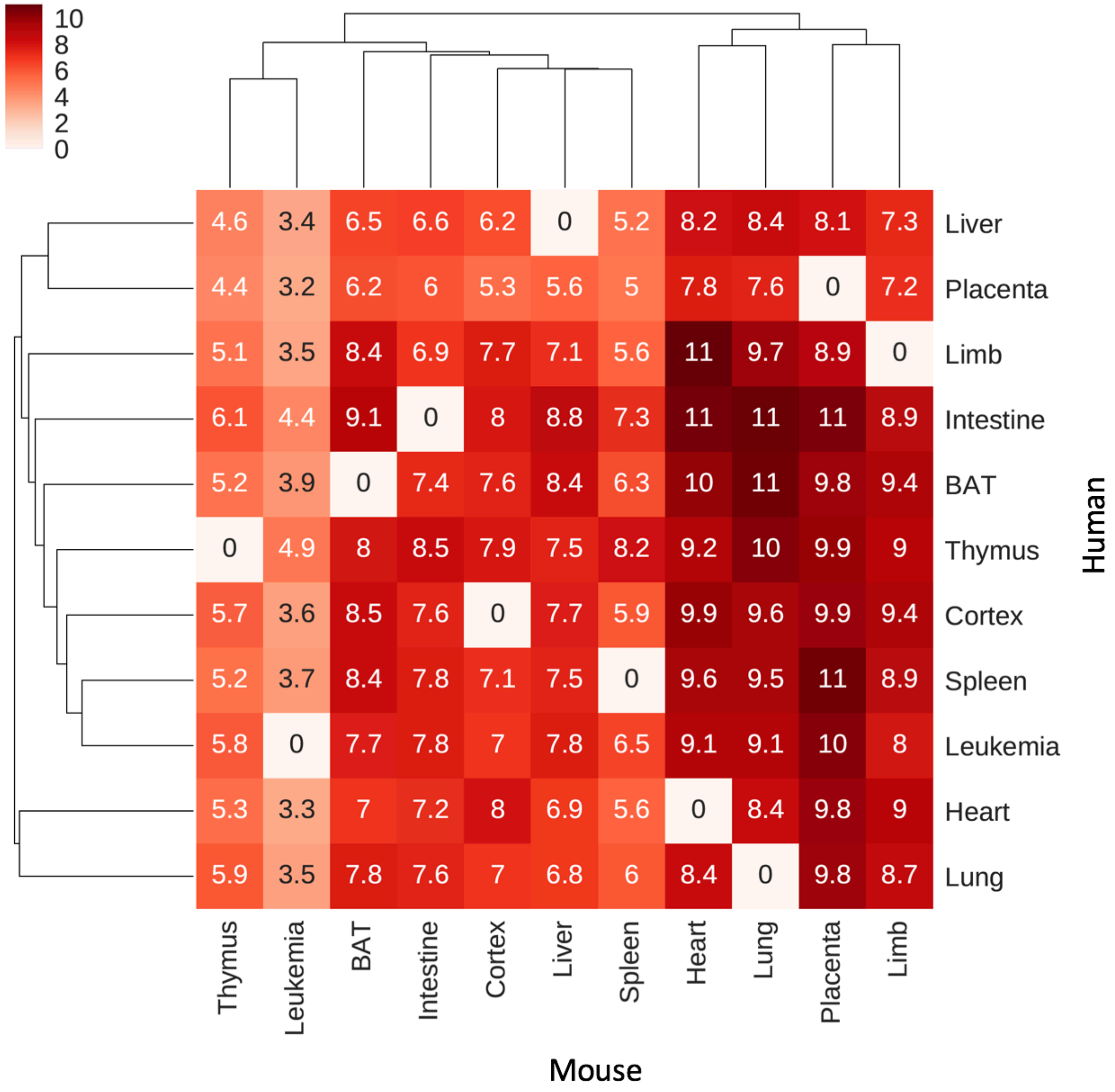

Figure S2. Percentages of the RPEs for each pair of tissues. Percentages of enhancers categorized as RPEs for each pair of tissues with respect to the total number of enhancers in each human tissue.

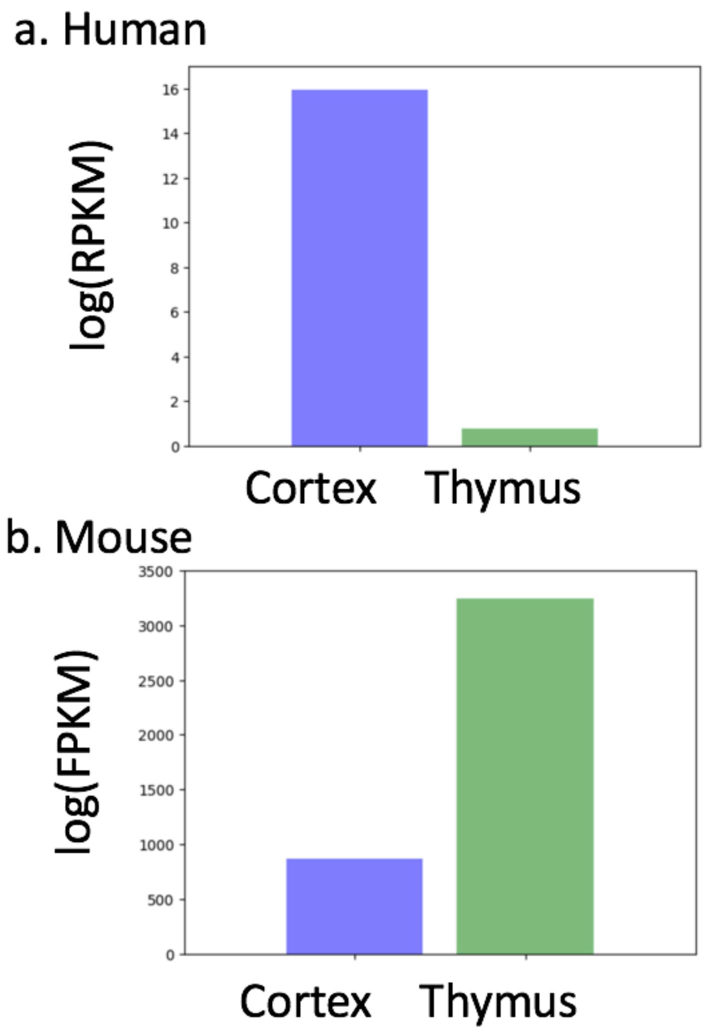

Figure S3. Log expression of THY1 gene. A RPE was found approximately at 9 kbp upstream of THY1. The RPE corresponds to the reprogramming to mouse thymus and human cortex. Top panel shows expression in human cortex and thymus as normalized log of Reads Per Kilobase of transcript per Million mapped (RPKM) reads. Bottom panel shows expression in mouse cortex and thymus normalized log of Fragments Per Kilobase of transcript per Million mapped (FPKM) reads. FPKM and RPKM, are similar but FPKM is calculated by counting fragments, not reads. Due to differences in protocols comparison between human and mouse tissues is not recommended.

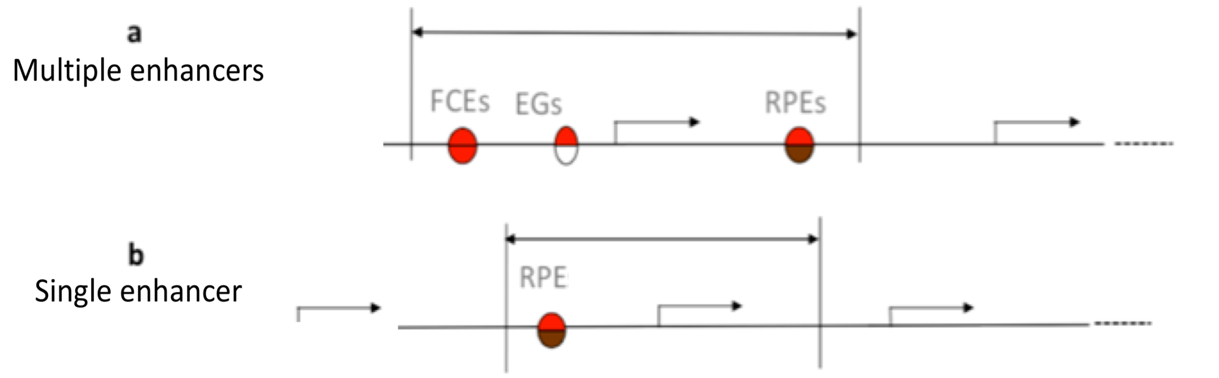

Figure S4. Distribution of reprogrammed enhancers. a. A cartoon showing FCEs, RPEs, and EGs within the locus of a gene. **b.** Case where only one RPE is within the locus of a gene.

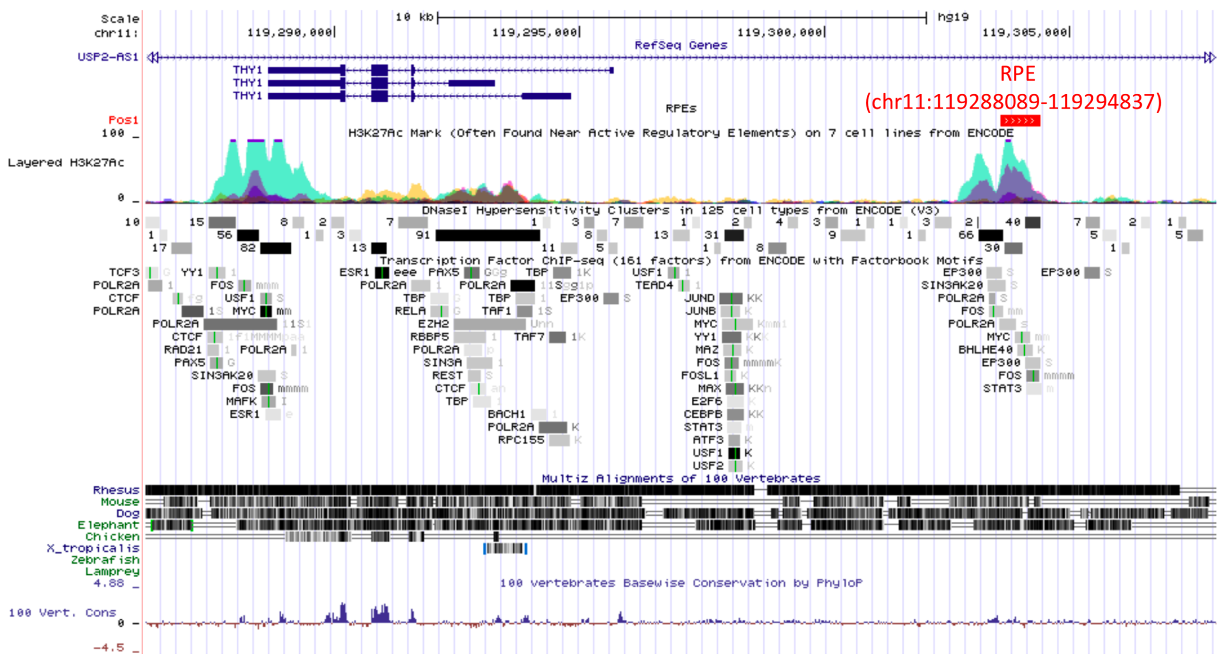

Figure S5. Genome browser showing the location of a RPE (to mouse thymus and human cortex) in human (hg19) within the locus of gene THY1. One reprogrammed enhancer (red rectangle top right) was found within the locus of gene THY1. Layered H3K27ac, DNaseI hypersensitivity clusters, TFs and conservation are shown as tracks below the RPE.

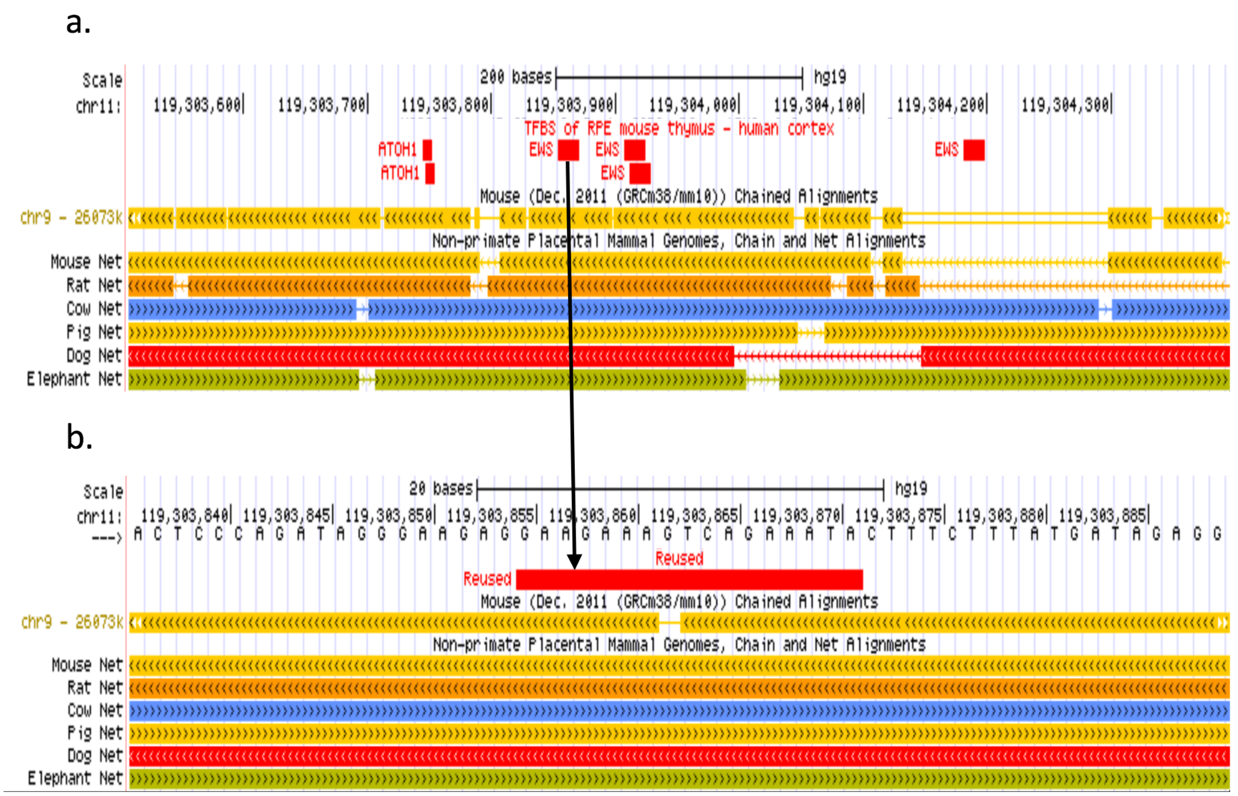

Figure S6. Genome browser showing the location of 6 TFBS of a RPE (to mouse thymus and human cortex) in human (hg19) within the locus of gene THY1. a. Red rectangles are sites for EWS and ATOH1 transcription binding sites. b. EWS TFBS categorized as reused site.

Table S1. **The number of reprogrammed enhancers for 11 pairs of tissues**. The index (m.) corresponds to mouse while the index (h.) corresponds to human, e.g., m.thymus-h.bat represents enhancers reprogrammed to mouse thymus and human BAT (brown adipose tissue).These sets are ordered by the number of enhancers for each human tissue.

m.leukemia-h.bat	1084	m.thymus-h.leukemia	866	m.leukemia-h.placenta	783
m.thymus-h.bat	1371	m.spleen-h.leukemia	885	m.spleen-h.placenta	1001
m.spleen-h.bat	1439	m.cortex-h.leukemia	941	m.thymus-h.placenta	1009
m.cortex-h.bat	1618	m.bat-h.leukemia	961	m.cortex-h.placenta	1053
m.liver-h.bat	1673	m.liver-h.leukemia	1003	m.liver-h.placenta	1103
m.limb-h.bat	1717	m.intestine-h.leukemia	1069	m.bat-h.placenta	1141
m.intestine-h.bat	1718	m.limb-h.leukemia	1100	m.limb-h.placenta	1239
m.heart-h.bat	1926	m.heart-h.leukemia	1108	m.intestine-h.placenta	1321
m.placenta-h.bat	2233	m.lung-h.leukemia	1200	m.heart-h.placenta	1333
m.lung-h.bat	2495	m.placenta-h.leukemia	1408	m.lung-h.placenta	1443
m.leukemia-h.cortex	809	m.leukemia-h.limb	1192	m.leukemia-h.spleen	640
m.spleen-h.cortex	955	m.spleen-h.limb	1482	m.thymus-h.spleen	770
m.thymus-h.cortex	1138	m.thymus-h.limb	1560	m.lung-h.spleen	936
m.heart-h.cortex	1217	m.bat-h.limb	1922	m.cortex-h.spleen	965
m.bat-h.cortex	1243	m.liver-h.limb	1927	m.heart-h.spleen	1010
m.liver-h.cortex	1247	m.cortex-h.limb	1954	m.liver-h.spleen	1093
m.limb-h.cortex	1328	m.intestine-h.limb	1991	m.bat-h.spleen	1094
m.intestine-h.cortex	1335	m.heart-h.limb	2418	m.limb-h.spleen	1104
m.lung-h.cortex	1379	m.placenta-h.limb	2454	m.intestine-h.spleen	1151
m.placenta-h.cortex	1780	m.lung-h.limb	2611	m.placenta-h.spleen	1577
m.leukemia-h.heart	887	m.leukemia-h.liver	1024	m.leukemia-h.thymus	525
m.spleen-h.heart	1093	m.thymus-h.liver	1298	m.spleen-h.thymus	671
m.bat-h.heart	1137	m.spleen-h.liver	1310	m.liver-h.thymus	689
m.liver-h.heart	1306	m.bat-h.liver	1422	m.bat-h.thymus	713
m.lung-h.heart	1306	m.cortex-h.liver	1506	m.intestine-h.thymus	724
m.thymus-h.heart	1347	m.intestine-h.liver	1663	m.cortex-h.thymus	729
m.cortex-h.heart	1501	m.heart-h.liver	1772	m.limb-h.thymus	770
m.intestine-h.heart	1503	m.limb-h.liver	1781	m.heart-h.thymus	795
m.limb-h.heart	1564	m.lung-h.liver	2047	m.lung-h.thymus	882
m.placenta-h.heart	2165	m.placenta-h.liver	2145	m.placenta-h.thymus	953
m.leukemia-h.intestine	556	m.leukemia-h.lung	693		
m.thymus-h.intestine	648	m.spleen-h.lung	734		
m.spleen-h.intestine	687	m.heart-h.lung	856		
m.liver-h.intestine	767	m.cortex-h.lung	954		
m.cortex-h.intestine	784	m.liver-h.lung	1018		
m.bat-h.intestine	785	m.bat-h.lung	1051		
m.limb-h.intestine	813	m.thymus-h.lung	1087		
m.heart-h.intestine	847	m.intestine-h.lung	1175		
m.lung-h.intestine	977	m.limb-h.lung	1211		
m.placenta-h.intestine	1082	m.placenta-h.lung	1523		

Table S2. Proportion of RPEs in locus binned by the number of enhancers in human liver

No. enhancers In locus	No. total enhancers	No. RPEs	% RPEs	Expression (RPKM)
1	3140	506	16	1.89
2	3532	624	18	2.25
3	3264	676	21	1.97
4	2888	732	25	2.20
5	2695	684	25	1.82
6	2178	567	26	2.14
7	2051	571	28	2.49
8	1608	402	25	1.96
9	1503	406	27	2.72
10	1480	402	27	2.55
11	1221	359	29	2.74
12	852	286	34	2.75
13	1001	304	30	2.77
14	798	231	29	2.67
15	810	263	32	2.73
16	768	218	28	3.16
17	731	257	35	4.77

Table S3. Proportion of RPEs in locus binned by the number of enhancers in human lung.

No. enhancers In locus	No. total enhancers	No. RPEs	% RPEs	Expression (RPKM)
1	2639	477	18	1.89
2	2750	621	23	2.63
3	2331	610	26	2.92
4	2108	633	30	2.91
5	1540	442	29	3.16
6	1920	576	30	2.99
7	1470	454	31	4.50
8	1288	377	29	3.59
9	1215	388	32	3.84
10	1260	361	29	3.38
11	1221	322	26	4.48
12	1020	324	32	3.85
13	819	229	28	5.77
14	938	279	30	5.41
15	705	230	33	4.86
16	688	218	32	5.04
17	527	173	33	4.91
